# Bioactive Properties of Carotenoids in Human Health

**DOI:** 10.3390/nu11102388

**Published:** 2019-10-06

**Authors:** Jaume Amengual

**Affiliations:** 1 Department of Food Sciences and Human Nutrition, University of Illinois Urbana Champaign, Urbana, IL 61801, USA; jaume6@illinois.edu; 2 Division of Nutritional Sciences, University of Illinois Urbana Champaign, Urbana, IL 61801, USA

**Keywords:** retinoids, carotenoids, bioactive compounds

## 1. Introduction

**Figure 1:**
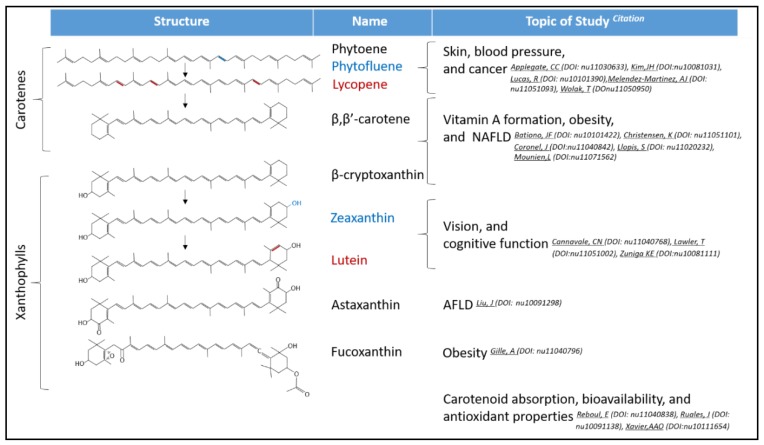
Topics covered by the Special Issue on carotenoids and human health. Colors present on carotenoids denote structural changes between similar carotenoids. Digital object identifier (DOI), non-alcoholic fatty liver disease (NAFLD), and alcoholic fatty liver disease (AFLD).

Research shows that certain bioactive compounds in our diet have beneficial effects on human health. Among these bioactive molecules, carotenoids are some of the most chemically and functionally diverse molecules in food, which encouraged us to prepare a Special Issue on “Carotenoids and Human Health”. The goal of this Special Issue is to provide a compilation of the recent advances on this important yet understudied research field. This Special Issue contains 11 original research articles, five literature reviews, one communication, and one discussion of cutting edge, peer-reviewed research papers (Figure 1).

Carotenoids are a group of pigments produced by all photosynthetic organisms. Due to their broad distribution in nature, it is logical that heterotrophic organisms have adapted and coevolved to utilize carotenoids for their own profit, such it occurs in birds, where the plumage pigmentation is perceived as a “health signal”, conditioning their reproductive success. In humans, some of these compounds serve as vitamin A precursor (pro-vitamin A carotenoids), and others are crucial for visual health (lutein), while most act as antioxidant molecules in lipid-rich environments. While there are over 650 different carotenoids described so far, the biological properties and therapeutic potential of these molecules has only been systematically studied for a handful of these molecules. 

Chemically, carotenoids are 40-carbon molecules with conjugated double bonds. They are divided into carotenes, composed of only carbons and hydrogen atoms, and xanthophylls, which also contain oxygen. Humans obtain carotenoids from their diets and accumulate them in relatively large amounts in tissues and plasma, where they play a variety of biological functions. On this note, Dr. Reboul’s discussion on carotenoid absorption provides a clear overview of this complex process, which will allow the reader to understand the biochemical and protein-mediated processes leading to carotenoid transport across the intestinal cell [1]. However, the first exposure to carotenoids seems to occur early during our development, as embryonic tissues express carotenoid-cleaving enzymes and transporters that allow the delivery of these compounds to the developing embryo [2,3]. Once born, breast milk will supply the developing child with the necessary carotenoids and retinoids. Xavier and colleagues studied if premature delivery affects the level of carotenoids in breast milk. They observed that in the case of premature birth, the total levels of carotenoids are decreased in the colostrum, with the exception of lutein, which in humans has a clear role in vision and possibly in brain development [4]. 

One of the most recognized properties of carotenoids is their antioxidant potential, as Ruales’ group indicates. These authors took a modern approach focused on the beneficial effects of the discarded parts of mangos, which are rich in carotenoids. Ruales’ work shows that mango skin and kennel are rich sources of bioactive compounds, and that some of these antioxidant molecules are not present in the pulp. These authors invites us to consider a better use of these fruit parts, which could also contribute to reduced waste production [5].

## 2. Bioactive Properties of Carotenes

Carotenes are one the first intermediates on carotenoid synthesis; therefore, these compounds are present in all photosynthetic organisms and highly abundant in our diet. Some of these early precursors such as phytoene and phytofluene are colorless, as they only contain three and five conjugated double bonds, respectively (Figure 1). As Dr. Melendez-Martinez’s review points out, these carotenes have been largely ignored for many years by the carotenoid field, a topic that recently has been gaining more interest. Since these carotenes accumulate in the skin, it is not a surprise that these enigmatic carotenes play a role in skin protection against UV light and are therefore involved in skin aging and health [6]. 

Further saturation of phytofluene will generate lycopene, another intermediate of carotenoid and xanthophyll biosynthesis, as well as one of the most abundant carotenoids in our plasma and tissues. Unlike phytoene and phytofluene, lycopene is red, and it is predominantly found in tomato and tomato products. Several studies show that the consumption of foods rich in lycopene, and tissue levels of this acyclic carotene, are associated with a low incidence certain types of cancer and cardiovascular disease. Indeed, Applegate and colleagues prepared complete systematic review focused on the preclinical studies studying lycopene and the androgen axis that controls prostate cancer. The authors concluded that the scientific evidence published to date shows that lycopene has an inhibitory effect of this hormonal axis, which could explain the positive properties of lycopene on this devastating disease [7]. Next, Kim and colleagues studied the relationship of gastric cancer with dietary consumption of different carotenoids. In a large cohort of patients (415 patients suffering from gastric cancer and 830 control individuals), the authors showed that the consumption of lycopene-containing foods (tomatoes and their derivatives) has an inverse correlation with the development of gastric cancer [8].

As mentioned above, some studies have also shown a correlation between lycopene and the development of heart disease, at least in part by affecting blood pressure. In another exciting human study, Wolak and colleagues evaluated whether pure lycopene or a tomato-based formulation named Tomato Nutrient Complex could reduce blood pressure. The authors showed that only their tomato-based formulation was effective at reducing systolic blood pressure. Interestingly, the authors observed an increase in the plasma levels of phytoene and phytofluene, together with lycopene, suggesting that these carotenes could be the mediators of the positive effects described in their study [9]. 

## 3. Bioactive Properties of Xanthophylls

The addition of oxygen groups to carotenes, mostly hydroxyl, ketone, and epoxide groups, gives rise to xanthophylls. Due to the variety of chemical substitutions and isomers, xanthophylls are the most diverse and unknown of the two big carotenoid subfamilies. While xanthophylls are generated by plants, ingested xanthophylls can be modified in animals by isomerization or reduction of these oxidized groups as they occur systemically in rodents [10] or in the macula lutea of primates and humans [11]. Depending on the number and type of oxygen groups, xanthophylls can present different chemical properties, but overall this group is more polar than their carotene precursors, a property that facilitates their intestinal absorption. 

Two of the most abundant carotenoids in our plasma are lutein and zeaxanthin. These xanthophylls are highly concentrated in our macula lutea, where they prevent photochemical damage. Many studies show that lutein supplementation, and to a lesser extent zeaxanthin supplementation, delays the development of age-related macular degeneration, a very common visual disorder in elderly. In Lawler’s review, however, the authors focused on the effect of these xanthophylls, and other dietary antioxidants, on the development of glaucoma. Based on the authors’ bibliographical research, a causal relationship between the risk of developing glaucoma and the consumption of antioxidants has not yet been established. However, as they point out for some preclinical studies, it is possible that lutein supplementation and zeaxanthin supplementation have some beneficial effects on this disease that deserve further study [12]. 

In recent years, researchers have been focusing on studying the role of lutein in cognition as this xanthophyll is preferentially accumulated in the brain. The contribution of Cannavale’s work to this topic shows that lutein plasma levels correlate with elevated hippocampal function in obese individuals [13]. Additionally, Zuniga’s paper establishes an association between total plasma carotenoid and inflammatory markers with cognitive function in cancer survivors. The authors observed that cancer survivors with low carotenoid plasma levels present higher rates of self-reported cognitive dysfunction [14]. Overall, these three articles contribute to understanding the positive functions of carotenoids, especially lutein, in vision and cognition. These effects could be a consequence of the specific accumulation of this pigment in neural tissues such as the brain and the eye. 

Xanthophylls are the most diverse group of carotenoids, but the biological properties of most of them are still understudied. This is the example of astaxanthin, a red carotenoid present in shrimps and other seafood. Interestingly, the production for commercialization as a nutritional supplement is one of the largest growing industries in the carotenoid field. This interest is based on its antioxidant properties, together with the relatively easy production, as this carotenoid is mostly obtained from algae, avoiding chemical synthesis [15]. Liu’s team examined the changes that occur in microbiota in response to astaxanthin supplementation and evaluated its effects in alcoholic fatty liver disease in mice. They observed that astaxanthin supplementation ameliorated ethanol-induced liver injury, and it is associated with the recovery of specific microbiome populations lost during ethanol supplementation [16]. Christensen’s work, however, focused on non-alcoholic fatty liver disease, which is one of the most common pathologies in developed countries and is deeply associated with obesity and other lipid-related disorders. They used data from a large human cohort to compare the prevalence of the disease by the level of the main carotenoids in diets and supplements, and in plasma. The authors observed that higher levels of most carotenoids studied, including those with a provitamin A activity, were associated with lower odds of developing non-alcoholic fatty liver disease [17]. 

Another interesting xanthophyll covered in this Special Issue is fucoxanthin. This carotenoid is present in edible seaweeds, and is largely consumed in Asia. Gille’s group evaluated the effects on obesity of a microalgae extract, rich in fucoxanthin, using mice as a preclinical model. The authors showed that this extract has beneficial effects on different adipose tissue parameters such as adipocyte size and gene expression markers. Interestingly, these effects seem to be slightly different than those observed with pure fucoxanthin in cultured adipocytes, indicating that it is possible that this carotenoid must be metabolized in the liver or intestine to exert its action, or that other components of the extract contribute to the positive effects of this carotenoid [18]. 

## 4. Carotenoids as Pro-Vitamin A Precursors

While carotenoids play various roles in human health, it is undeniable that the most important function of these compounds is the production of vitamin A. Only carotenoids with an unsubstituted β-ionone ring have pro-vitamin A activity, and the most important of these carotenoids, and the most abundant of them in our diet and tissues, is β, β’-carotene (β-carotene). Vitamin A is a potent gene regulator controlling the expression of nearly 700 genes. Additionally, vitamin A is required for vision, as retinal is the visual chromophore in mammals. One of the most interesting roles of β-carotene and vitamin A is the role they play in lipid metabolism and obesity. In this line, Coronel’s group provides a bibliographical review of the effects of β-carotene in obesity. These authors divide their review into cell culture, animal, and human studies to dissect the main findings related to β-carotene and vitamin A over the past years, and use their expertise in the field to provide guidelines and considerations for future carotenoid researchers interested in joining this exciting field [19]. On the same line, Mounien summarizes the latest findings on the effect of different carotenoids on adipose tissue physiology, and the role of these compounds in the brain in relationship with obesity, proposing a provocative role of these molecules on the control of food intake, inflammation, and adipokine secretion [20]. 

Continuing with obesity, Llopis et al. studied the effect of β-cryptoxanthin, another provitamin A carotenoid, using *C. elegans* as a model. The authors showed that worms accumulated β-cryptoxanthin, and that this compound reduced oxidative stress. Additionally, the authors show that β-cryptoxanthin reduces lipid droplet accumulation and alters energy metabolic pathways [21], similarly to what was observed in white adipose tissue of β-carotene-fed mice [22]. 

As Coronel’s review points out, the main transcriptionally active form of vitamin A is retinoic acid [19]. Under normal conditions, this metabolite is present in small quantities in tissues, and its quantification is challenging [23,24]. Lucas and collaborators quantified the presence and different isoforms of key carotenoids and retinoids, including all-trans retinoic acid, in plasma of patients affected with atopic dermatitis in comparison to healthy patients. This skin disorder is very common in children in Western societies, and can become chronic in adults. The exact causes of atopic dermatitis are not fully understood, but this disorder is characterized by hyper-reactivity of the immune system and skin to certain, otherwise harmless, antigens. The author shows that patients with atopic dermatitis present an overall reduction of key carotenoids such as lutein and β-carotene, accompanied by a reduction in all-trans retinoic acid. These results indicate that low levels of carotenoids/retinoids, together with an altered ratio of some of their isomers, could be associated to the maintenance or the development of atopic dermatitis [25]. 

Vitamin A deficiency is the single most important cause of childhood blindness in developing countries, and is responsible for millions of deaths due to immune-related disorders. Therefore, the early identification of this deficiency is crucial to prevent life-lasting consequences or even death. This exciting topic is the main focus of Bationo’s paper, where the authors correlate the intake and plasma levels of pro-vitamin A carotenoids, and their association with vitamin A status among children in Burkina Faso [26]. 

## 5. Conclusions

Carotenoids and retinoids are part of our diet and present in our tissues and plasma. Understanding the implication of these bioactive molecules on human health will contribute to providing better nutritional guidance. Thanks to the work published in this Special Issue, we hope to increase the interest of this exciting field of research among researchers, and to also inform the general public of the importance of carotenoids on human health.
